# Advanced Echocardiographic Analysis in Medium-Term Follow-Up of Children with Previous Multisystem Inflammatory Syndrome

**DOI:** 10.3390/children9060917

**Published:** 2022-06-18

**Authors:** Massimo Garbin, Irene Raso, Alessandra Piersanti, Laura Gianolio, Annalisa De Silvestri, Valeria Calcaterra, Carla G. Corti, Luisa F. Nespoli, Sara Santacesaria, Giulia Fini, Dario Dilillo, Gianvincenzo Zuccotti, Savina Mannarino

**Affiliations:** 1 Pediatric Cardiology Unit, “Vittore Buzzi” Children’s Hospital, 20154 Milan, Italy; massimo.garbin@asst-fbf-sacco.it (M.G.); carla.corti@asst-fbf-sacco.it (C.G.C.); luisa.nespoli@asst-fbf-sacco.it (L.F.N.); santacesaria.sara@asst-fbf-sacco.it (S.S.); giulia.fini@asst-fbf-sacco.it (G.F.); savina.mannarino@asst-fbf-sacco.it (S.M.); 2 Department of Woman and Child Health and Public Health, Fondazione IRCCS Policlinico Universitario Agostino Gemelli, 00168 Rome, Italy; piersanti.ap@gmail.com; 3 Pediatric Department, “Vittore Buzzi” Children’s Hospital, 20154 Milan, Italy; laura.gianolio@unimi.it (L.G.); valeria.calcaterra@unipv.it (V.C.); dario.dilillo@asst-fbf-sacco.it (D.D.); gianvincenzo.zuccotti@asst-fbf-sacco.it (G.Z.); 4 Biometry and Clinical Epidemiology, Scientific Direction, Fondazione IRCCS Policlinico San Matteo, 27100 Pavia, Italy; a.desilvestri@smatteo.pv.it; 5 Department of Internal Medicine, University of Pavia, 27100 Pavia, Italy; 6 Department of Biomedical and Clinical Science, University of Milan, 20157 Milan, Italy

**Keywords:** COVID-19, MIS-C, strain, speckle tracking echocardiography

## Abstract

Multisystem inflammatory syndrome in children (MIS-C) is a severe hyperinflammatory disease related to SARS-CoV2 infection, with frequent cardiovascular involvement in the acute setting. The aim of the study was to evaluate the cardiac function at 6 months. Thirty-two patients diagnosed with MIS-C were enrolled and underwent advanced echocardiogram at discharge and at 6 months. According to the left ventricular ejection fraction (LVEF) at admission, the patients were divided into group A (LVEF < 45%) and group B (LVEF ≥ 45%) and the follow-up results were compared. At discharge, all patients had normal LV and RV systolic function (LVEF 61 ± 4.4%, LV global longitudinal strain −22.1%, TAPSE 20.1mm, s’ wave 0.13m/s, RV free wall longitudinal strain −27.8%) with normal LV diastolic function (E/A 1.5, E/e’ 5.7, and left atrial strain 46.5%) and no significant differences at 6 months. Compared to group B, the group A patients showed a reduced, even if normal, LV global longitudinal strain at discharge (−21.1% vs. −22.6%, *p*-value 0.02), but the difference was no longer significant at the follow-up. Patients with MIS-C can present with depressed cardiac function, but if treated, the cardiac function recovered without late onset of cardiac disease. This favorable result was independent of the severity of acute LV dysfunction.

## 1. Introduction

Multisystem inflammatory syndrome in children (MIS-C) is a rare but severe hyperinflammatory disease that affects pediatric patients, typically 3–6 weeks after SARS-CoV2 infection [1,2]. Even if the pathophysiological mechanism of the disease is still unclear, the proposed mechanism is a post-infectious phenomenon mediated by IgG antibodies enhancement in genetically susceptible children with a possible role of oxidative stress [3,4].

The disease affects many organs, cardiac involvement is one of the commonest, up to 80%, and it can influence the clinical presentation, the need for intensive care, the management, and the outcomes. Although there is not a consensus regarding the proper immunomodulatory treatment [5,6], immunoglobulin and corticosteroids remain the cornerstone of therapy and after their administration, MIS-C patients swiftly recover with mortality between 0% and 3%, at least in the high-income countries’ literature [7,8].

In the acute phase, the cardiovascular system is generally affected by mild to moderate left ventricle (LV) systolic depression, and only one-third of patients present with severely reduced LV ejection fraction (EF < 30%). Cardiogenic or vasodilatory shock can occur in up to half of the MIS-C diagnoses. Mechanisms of myocardial dysfunction are still not clear, with some evidence of post-infective immune-mediated myocarditis [9] detected with cardiac magnetic resonance (CMR). The initial cardiologic reports show an early cardiac function improvement within 7–10 days from the therapy, detected by echocardiogram, and confirmed by the early short-term results of CMR which show overall normal findings, except for a small subgroup of patients [10].

Up to now, most papers describe only the acute and early follow-up myocardial function with traditional echocardiographic techniques [7,9,11,12,13,14,15], but the ability to detect LV dysfunction can be implemented by advanced techniques, such as global longitudinal strain (GLS). Speckle tracking echocardiography is an angle-independent measurement that has been used to evaluate deformation and quantitatively characterize LV function. It is more sensitive than normal EF in the detection of subclinical LV myocardial dysfunction and its ability to predict ventricular dysfunction has already been demonstrated in patients with myocarditis, even with a preserved ejection fraction at the traditional evaluation [16]. The first reports of these advanced data [9,10,11,17] documented a higher power to detect patients with some degree of residual LV dysfunction in the short-term follow-up.

Because LV function is an important prognostic determinant of cardiopulmonary pathologies in children, there is an overall agreement around the need for further follow-up dates to understand the risk of permanent dysfunction in MIS-C. To our best knowledge, there were no published data regarding long-term cardiac outcomes of patients with MIS-C in terms of monitoring coronary artery dimensions, biventricular function, and myocardial involvement with advanced echocardiographic techniques.

In this study, the cardiologic follow-up data of our previously published cohort of MIS-C patients were reported [12]. The primary aim of the study was to evaluate if there was any cardiac dysfunction in patients with MIS-C at the 6-month follow-up, using both traditional and advanced echocardiogram techniques. We also compared patients who experienced a significant cardiac dysfunction in the acute phase of illness with those without cardiac involvement. A long-term follow-up may be warranted to better define the cardiac damage caused by hyperinflammatory syndrome.

## 2. Materials and Methods

This is a retrospective, single-center study performed at the Pediatric Department of the Children’s Hospital, Vittore Buzzi, Milan. We included all children and adolescents who met the criteria of MIS-C diagnosis according to the Center for Disease Control and Prevention (CDC) [18], who were admitted to our hospital from the 1st of November 2020 to the end of February 2021. At admission, all patients were tested for SARS-CoV2 acute infection by RT-PCR nasopharyngeal swab and for serological assay targeting a recombinant nucleocapsid N-spike S protein of SARS-CoV2. All patients underwent complete pediatric evaluation, blood tests (routine and those included in CDC definitions), and organ damage evaluation. Patients were treated accordingly to our multidisciplinary protocol, which was written in October 2020 on the basis of the evidence in the literature [19,20]. In Table 1, data from the acute phase were summarized, as previously published [12]. The study was conducted accordingly to the guidelines of the Declaration of Helsinki, and approved by the Buzzi Hospital review board, with all participants and parents informed.

The first cardiologic evaluation was carried out before IVIG administration. LVEF was calculated with Simpson’s biplane method with Philips Affinity 70 or Vivid S5 GE Healthcare. Accordingly, to the degree of acute LV function, we divided MIS-C patients into 2 groups: group A, patients with moderate to severe depression of LVEF (LVEF < 45%), and group B, patients without or with mild signs of cardiac involvement (LVEF ≥ 45%). All patients were closely followed by the cardiologists. At discharge and at 6 months, all patients underwent standard and advanced transthoracic echocardiography, recorded with Vivid S5 GE Healthcare by a single expert pediatric cardiologist (M.G.). Standard echocardiographic measurements were made, accordingly to guidelines [21], including M-mode of the LV [22], left ventricular ejection fraction with Simpson’s biplane formula, diastolic LV function with early and late mitral inflow peak velocities, tissue doppler images (TDI) of septal and lateral annular peaks, and LV mass indexed for body surface area (BSA) and for height [23]. For the right ventricle, the M-Mode of the tricuspid annular plane systolic excursion (TAPSE) and TDI of the annular tricuspid peak velocity. Z-score indexed for BSA was calculated. Coronary arteries’ involvement was evaluated with attention in accordance with guidelines [24]. Coronary Z-scores were reported using the Boston Z-score system (normal <2, dilatation ≥ 2 to <2.5, aneurysm ≥ 2.5). The advanced analysis included a measure of myocardial deformation, with a 2-dimensional speckle tracking analysis. Analysis was performed offline with independent software (TOMTEC Imaging Systems GmbH, www.tomtec.de/technical-docs, accessed on 1 April 2022). The LV endocardial borders were automatically traced, after the manually setting of the mitral annulus points and the apex. The trace was manually adjusted only if needed. GLS was calculated as the average of the peak systolic longitudinal strain from all LV segments from the 4-, 2-, and 3-chamber images. We considered age-dependent normal values with 95% confidence interval (accordingly to published vendor normal range): 0–1 year old −18.7% (95% CI, −20.8% to −16.7%), 2–9 years old −21.7% (95% CI, −23.0% to −20.5%), 10–13 years old from −20% (95% CI, −20.8% to −19.1%), and 14–21 years old −19.9% (95% CI, −20.6% to −19.2%) [25]. Values above the lower limit were considered borderline values and values > −16% as a reduced systolic function. From the 4-chamber view, peak global left atrial reservoir strain (LAS) was recorded. LAS is an age-dependent value [26], and there were no normal reference values for children. The cut-off that we used (LAS > 40%) was based on the mean normal published range [11,27,28,29]. For the RV function, the peak longitudinal strain of the right ventricular free wall (RVFWLS) was measured from four chamber images, and values > −29.03% (95% CI, −31.52% to −26.54%) were considered the normal mean values in children [30].

Statistical analyses were performed using Stata 16 (StataCorp, College Station, TX, USA). Categorical variables are described as count and percentage; quantitative ones as mean and standard deviation (sd) if normally distributed (Shapiro–Wilks test), as median and interquartile range (IQR) otherwise. Comparisons of quantitative variables at 6 months and at discharge were made with paired *t*-test or non-parametric Wilcoxon test, comparisons between groups with independent *t*-test and Mann–Whitney test.

## 3. Results

### 3.1. Demographic and Biochemical Data of the Population

We collected 32 consecutive patients with diagnoses of MIS-C, all with positive IgG serology for SARS-CoV2. According to cardiac function, ten patients (31.25%) were included in group A with myocardial dysfunction (median LVEF 33.5% ± 7.3) and 22 (68.75%) in group B without or with only mild cardiac involvement (median LVEF 55.8% ± 6.1). Our population was uniform for the baseline characteristics, without significant differences between the groups. In the acute phase, all patients had laboratory data showing a hyperinflammatory state. C-reactive protein was elevated in all patients (median 200.8 mg/L (119.48–279.3)), with significantly higher median values for group A (283 mg/L (261–308)) compared with group B (178 mg/L (92–241)), *p*-value 0.008. Cardiac biomarkers were significantly higher in group A (troponin T 72 ng/L (40–243)) and NT-pro-BNP 14,825 ng/L (11,340–17,810)) than in group B (troponin T 22ng/L (8–49)) and NT-pro-BNP 5921 ng/L (1114–11,243)), *p*-value 0.01 for both variables. All patients (100%) were treated with IVIG at the time of diagnosis. Treatment with steroids (methylprednisolone) was added for 30 patients (93.8%): 15 (46.9%) received the intermediate-high dose (10–30 mg/kg iv pulse for 3 days), and 15 (46.9%) the lower dose (2 mg/kg iv for at least 5 days), depending on the severity of the disease at the presentation, followed by gradual tapering. None of our patients received a second dose of IVIG. Low-molecular-weight heparin was prescribed for 29 patients (91%), of whom seven (22%) received a therapeutic anticoagulation dosage. All patients were discharged with low-dose aspirin and 93.8% with oral corticosteroid. A detailed discussion of the acute phase data was out of the purpose of this study, and it was previously reported in another paper [12].

### 3.2. Advanced Echocardiographic Evaluation in the Overall Population

All patients underwent a complete cardiac evaluation within 7 days from the discharge and after 6 months. We reported a significant mean increase in BSA from discharge to 6 months follow-up (1.17 kg/m^2^ (±0.37) vs. 6 months (1.22 kg/m^2^ (±0.37), *p*-value < 0.001).

Table 2 showed echocardiographic data. At discharge, all patients returned to normal LV and RV systolic function (mean LVEF was 61 ± 4.4%, LV GLS −22.1%, TAPSE 20.1 mm, s’ wave 0.13 m/s, RVFWLS −27.8%) with normal LV diastolic function (E/A 1.5, E/e’ 5.7 and LAS 46.5%) and normal indexed wall thickness, diameters, volumes, and mass (EDD (end-diastolic diameter) Z-score 0.41, IVSd (interventricular septum diastolic) Z-score 0.56, PWd (posterior wall diastolic) Z-score 0.27, EDVi (indexed) 54.1 mL/m^2^, LV mass 83 g, LV mass indexed for BSA 68.4 g/m^2^, and LV mass indexed to height 34.2 g/m^2^). All patients had normal coronary artery diameters.

The persistence of normal values was confirmed at the 6 months follow-up without statistically significant differences among most of the echocardiographic variables. The only significant parameters were the improvement of RV s’ wave (0.14 m/s at 6 months vs. 0.13 m/s discharge, *p*-value 0.049) and the increase in LVEF (63.3% vs. 61.0%, *p*-value 0.002) but without difference in LV GLS (−21.1% vs. −21.1%, *p*-value ns).

Interestingly, LV diameters, volumes, and masses decreased from discharge to 6 months follow-up, even with different degrees of statistical significance: EDD was 41.2 mm (±6.0) at the discharge vs. 40.1 mm (±6.0) at 6 months (*p*-value ns), EDV was 65.3 mL (±26.7) vs. 63.2 mL (±23.9) (*p*-value ns); while the end-diastolic volume indexed, LV mass, and LV mass indexed reached the statistical significance (EDVi 54.1 mL/m^2^ (±9.4) vs. 50.8 (±6.9); LV mass 83.0 g (±34.4) vs. 77.7 g (±33.6) and LV mass/BSA 68.4 g/m^2^ (±13.8) vs. 63.5 g/m^2^ (±11.3), with *p*-value 0.017, 0.002, and 0.001, respectively).

### 3.3. Advanced Echocardiographic Evaluation: Group A vs. Group B

**Figure 1:**
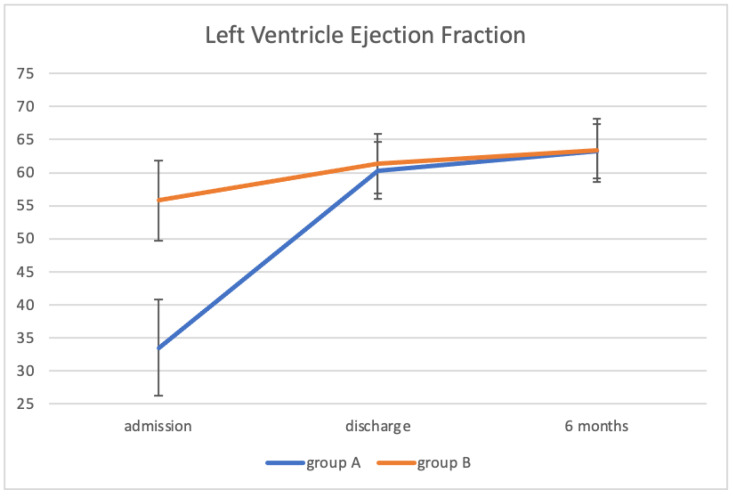
Left ventricle ejection fraction trends from the admission to the 6-month follow-up. Ejection fraction improved at the discharge and remains within the normal range at the medium-term follow-up.

Table 3 showed the echocardiographic results divided into the two prespecified groups. Interestingly, at discharge evaluation, all cardiac parameters normalized, regardless of the degree of LV dysfunction in the acute phase (Figure 1). Compared to group B, the group A patients showed a reduced LV GLS (group A−21.1% vs. group B−22.6%, *p*-value 0.02) even if both values were normal. The difference was no more detectable at the 6 months evaluation (−22.3% vs. −22.1%, *p*-value ns). There was also a slight reduction in RV systolic function measured as RVFWLS, the borderline for statistical significance (group A −25.8% vs. group B −28.7%, *p*-value 0.05). All the other parameters were comparable between the two groups both at discharge and at 6 months.

## 4. Discussion

MIS-C is a rare and highly severe disease that affects children previously infected by SARS-CoV2. The cardiovascular system is one of the main systems involved and it is the major determinant of the acute management and prognosis. Many reports documented a good short-term outcome, but little is known about medium- and long-term follow-ups, and to date, there is only one pediatric paper reporting 3–6-month outcomes, although without insight into the cardiologic aspects [31]. According to the available literature, the LVEF evaluated by a traditional echocardiogram tends to normalize within 7–10 days after acute treatment. There is an increased interest in the impact of strain in clinical practice, thanks to its ability to identify subtle left ventricular dysfunction, even in those with normal EF. Strain can indicate a higher risk for adverse acute clinical course and persistent subclinical left ventricular dysfunction [32]. At the beginning of the COVID-19 pandemic, Matsubara et al. demonstrated both LV systolic and diastolic dysfunction in MIS-C patients in the acute phase, and most of the patients restored a normal LVEF during the first week of treatment, but with a lower median GLS and a mild diastolic dysfunction if compared with normal children [11]. Similar data were reported by the Padua group in which the acute advanced echocardiographic analysis showed a reduction in acute-phase LV GLS (mean value −17%). Interestingly, they were able to compare the echocardiographic data with the in-hospital cardiac MRI (CMR) in which they found a 35% of late gadolinium enhancement (LGE) supporting the hypothesis of a post-viral immune-mediated cardiac damage [9]. In our previous report, the association between the severity of cardiac dysfunction and the extent of the illness was demonstrated. We now reported the echocardiographic 6 months follow-up data of our previously published cohort of patients [12], where all patients were evaluated with both traditional and advanced echocardiographic techniques. The basic assumption of our paper is that speckle tracking analysis is more sensitive than traditional echocardiography in detecting myocardial injury [33]. The results of the study proved that all the echocardiographic parameters were normal at discharge and remained normal at the 6-month follow-up, without statistically significant differences. At discharge, the LVEF was slightly reduced compared to those measured at 6 months, but that finding was not supported by the GLS analysis. GLS is considered a more robust measure of LV systolic function than EF, because it is calculated as the average of the longitudinal strain from all LV segments, while the EF is a geometrically derived and manually traced parameter, and more prone to errors. Relatedly, RV systolic function was in the normal range at discharge and at medium-term follow-up. However, there was an upward trend in all the RV measured variables, which did not reach a statistical significance, except for s’ wave. This finding suggested that from discharge to 6 months there is an overall tendency towards the improvement of the RV systolic function. With regard to the diastolic function, a previously published report showed a markedly reduced diastolic function in the acute phase of MIS-C patients with cardiac injury, if measured by LAS (mean MIS-C 20.5% vs. normal control 40.5%). Furthermore, they demonstrated that LAS was the strongest parameter associated with myocardial damage. LAS is raising interest as an excellent parameter with easy feasibility that can detect LV diastolic alterations and elevated LV filling pressure [34,35]. In our cohort, we documented the absence of diastolic dysfunction both at the early- and the medium-term follow-up, with standard and advanced parameters. If LAS can be considered a strong parameter of myocardial damage, we can assume that after the acute phase of illness there is a good recovery of function, without significant residual injury. 

Coronary involvement in MIS-C is highly debated, due to a significant difference in the incidence of dilation in different studies, ranging from nearly 0% up to 25% [11,12,13,36,37,38]. The wide range could be possibly explained by some overlap with Kawasaki disease, by the delay in the diagnosis and therapy, and by a different susceptibility related to the ethnic group or to SARS-CoV2 variants. Independently the acute coronary damage, there is consensus regarding the need for further medium-term results. In our cohort, only one patient experienced a transient coronary artery ectasia in the acute phase with normalization in the first week of illness and stable diameters in the follow-up period. In our cohort, coronary arteries were quite spared by the disease in the acute phase and remained within the normal range over the follow-up, without neither discrete nor segmental aneurysms. Those findings are in line with the first report of Verdoni et al., Belhadjer et al., and with the U.S. report of Matsubara [7,11,13] and suggested that in those without or with only mild coronary artery involvement, there were neither a tardive dilation nor progressive stenosis in the follow-up. We observed a trend towards a reduction in LV volumes, diameters, and masses. This trend could be ascribed to a reduction in fluid overload and to the healing of the myocardium from the edema. At admission, we noticed a general tendency to volume overload with high central venous pressure and the need for diuretics to force diuresis and unload ventricles. Possibly, diameters and volumes measured at discharge, even if within the normal range are still influenced by the acute phase overload and they took time to restore to their right dimensions. The mass variations could reflect the reduction in diameters and the resolution of myocardial wall edema.

A further analysis was performed by dividing the population into the two prespecified groups. The two groups were homogeneous for all the echocardiographic parameters. Very interestingly, the only significant difference was a reduced, even if normal, mean LV GLS in group A compared to group B. The difference was detectable at discharge, but no more at the medium-term follow-up. The hypothesis is that in the group with more significant cardiac involvement, some degree of systolic impairment residues at the early follow-up despite normal LVEF, but it completely recovers at the 6-month evaluation. These findings supported the concept that cardiac involvement in MIS-C is a reversible and transient condition. In our cohort, all patients, even those with acute severe cardiac involvement, completely heal in a relatively short time frame and at 6 months no residual differences between the groups can be found. The pathophysiological mechanism of cardiac dysfunction is still not completely elucidated. Although, in most cases, cardiac dysfunction seems the result of the hyperinflammatory state and the cytokine storm that cause myocardial stunning and perivascular edema, rather than a direct myocyte injury or an immune-mediated viral trigger. The hypothesis is supported by the disproportion between myocardial damage and the relatively low increase in troponin serum level, while there is a direct correlation between the severity of heart involvement and NTproBNP and inflammatory markers [10,39]. Trisha P. et al. showed a significant difference in time to recovery of LVEF comparing classic myocarditis and myocarditis linked to MIS-C (70% vs. 93%) and a significant difference in the needed heart failure medications at three months post-discharge (only 2.2% in MIS-C vs. 45% in classic myocarditis). Moreover, the peak of troponin was highest in the classic myocarditis group, whereas the MIS-C myocarditis group had the highest recorded brain natriuretic peptide [40].

This theory can be supported by the relatively low percentage of positive CMR with few cases of fibrosis and LGE compared to the numbers of cardiac dysfunction, even in the early follow-up [9,14,41,42].

Limitations. This is a single-center study, with a retrospective design and a small sample size which limit the strength of the study. Furthermore, the study did not include the advanced echocardiographic evaluation of the acute phase before the administration of the therapy. The analysis of strain at admission could have added important information, but the need to minimize exposure of the cardiologist and the use of a portable and less performing echo machine did not allow further evaluation. In addition, the clinical instability of patients often required quick evaluations, followed by intensive care and treatment. The first-line treatment with IVIG and corticosteroids allowed a quick clinical improvement and a restitutio ad integrum of the cardiac function; however, we are aware that the standard of care in MIS-C is still debated, and we cannot be sure that recovery can occur also without treatment.

## 5. Conclusions

MIS-C is a rare and potentially fatal novel disease, in which the heart and the cardiovascular system are frequently involved. We found that despite significant cardiac dysfunction in the early stage, all the traditional and advanced echocardiographic parameters were normal at discharge and at 6 months follow-up. We described an initially lower mean value of LV GLS in the group that experienced a significant cardiac injury in the acute phase of illness, but the difference was no more detectable at the follow-up.

Although patients with MIS-C myocarditis can present with severely depressed cardiac function, they completely recovered and cardiac function normalized. We did not record any late-onset of cardiac disease at the 6-month follow-up. However, longer monitoring will be necessary to provide further insight into the disease.

## Figures and Tables

**Table 1:** Baseline characteristics of the population, according to the degree of compromised myocardial function.

Baseline Characteristics	Total
Patient numbers (*n*)	32
Age (years), median (IQR)	10 (7–13)
Gender	
Male, *n* (%)	24 (75)
Female, *n* (%)	8 (25)
Overweight, *n* (%)	4 (12.4%)
RT-PCR positive, *n* (%)	2 (6.3)
Serology-positive, *n* (%)	32 (100)
Fever, *n* (%)	32 (100)
Fever duration (days)	6
Gastrointestinal, *n* (%)	30 (94)
Shock, *n* (%)	8 (25)
Neurological, *n* (%)	5 (16)
Hospital stay (days)	13
Pediatric Intensive Care Unit admission, *n* (%)	22 (69)
Pediatric Intensive Care Unit stay (days)	2
Noninvasive ventilation, *n* (%)	12 (37.5)
Ventilation (days)	2.5
Inotropic support, *n* (%)	11 (34.4)

Modified from Savina M et al. [12].

**Table 2:** Comparison between early (discharge) and late (6 months follow-up) echocardiographic data. Data are expressed in mean (± standard deviation). Z-scores are expressed in media (IQR) Legend. BSA (body surface area), EDD (end-diastolic diameter), EDV (end-diastolic volume), EDVi (end-diastolic volume indexed for body surface area), EF (ejection fraction), IVSd (interventricular septum diastolic), LAS (left atrium strain), LV GLS (left ventricle global longitudinal strain), ns (not significant), PWd (posterior wall diastolic), RVFWLS (right ventricle free wall longitudinal strain), TAPSE (tricuspid annular plane systolic excursion).

Echocardiographic Variables	Discharge (*n* = 32)	At 6 Months (*n* = 32)	*p*-Value
**RV systolic function**			
TAPSE (mm)	20.1 (±2.1)	20.7 (±1.9)	ns
s wave (m/s)	0.13 (±0.02)	0.14 (±0.02)	0.049
RVFWLS (%)	−27.8 (±3.9)	−28.1 (±3.9)	ns
**LV systolic function**			
EF (%)	61.0 (±4.4)	63.3 (±4.5)	0.002
LV GLS (%)	−22.1 (±1.8)	−22.1 (±1.9)	ns
**LV diastolic function**			
E/A	1.5 (±0.3)	1.7 (±0.4)	0.015
E/e’	5.7 (±1.3)	5.8 (±0.9)	ns
LAS (%)	46.5 (±7.9)	45.0 (±6.8)	ns
**LV measurements**			
EDD (mm)	41.2 (±6.0)	40.1 (±6.0)	ns
EDD Z-score median (IQR)	0.41 (−0.04–1.13)	0.39 (−0.01–0.86)	ns
IVSd (mm)	6.7 (±1.1)	6.7 (±1.1)	ns
IVSd Z-score median (IQR)	0.56 (−0.04–1.13)	0.19 (0.06–0.43)	ns
PWd (mm)	6.7 (±1.3)	6.6 (±1.1)	ns
PWd Z-score median (IQR)	0.27 (0.04–0.59)	0.27 (0.04–0.47)	ns
EDV (mL)	65.3 (±26.7)	63.2 (±23.9)	ns
EDVi (mL/m^2^)	54.1 (±9.4)	50.8 (±6.9)	0.017
LV mass (g)	83.0 (±34.4)	77.7 (±33.6)	0.0024
LV mass indexed for BSA (g/m^2^)	68.4 (±13.8)	63.5 (±11.3)	0.0012
LV mass indexed for heigh (g/m)	34.2 (±4.4)	30.9 (±3.1)	<0.001
**Coronary arteries dilation (Z-score > 2) (*n*)**	0	0	-

**Table 3:** Results of echocardiographic parameters in the early (discharge) and late (6 months follow-up) evaluation, divided into group A and group B. Variables are expressed in mean (± standard deviation) and median (and interquartile range) for Z-score values. Legend. BSA (body surface area), EDD (end-diastolic diameter), EDV (end-diastolic volume), EDVi (end-diastolic volume indexed for body surface area), EF (ejection fraction), IVSd (interventricular septum diastolic), LAS (left atrium strain), LV GLS (left ventricle global longitudinal strain), ns (not significant), PWd (posterior wall diastolic), RVFWLS (right ventricle free wall longitudinal strain), TAPSE (tricuspid annular plane systolic excursion).

Echocardiographic Variables	Group A, LVEF < 45% (*n* = 10)		Group B, LVEF ≥ 45% (*n* = 22)		*p*-Value	
	Discharge	At 6 Months	Discharge	At 6 Months	Discharge	At 6 Months
**RV systolic function**						
TAPSE (mm)	19.7 (±2.8)	20.7 (±2.1)	20.3 (±1.7)	20.6 (±1.9)	ns	ns
s wave (m/s)	0.14 (±0.03)	0.14 (±0.03)	0.13 (±0.02)	0.14 (±0.02)	ns	ns
RVFWLS (%)	−25.8 (±4.4)	−26.7 (±3.4)	−28.7 (±3.3)	−28.7 (±3.9)	ns (0.05)	ns
**LV systolic function**						
EF (%)	60.3 (±4.3)	63.3 (±4.1)	61.3 (±4.5)	63.4 (±4.8)	ns	ns
LV GLS (%)	−21.1 (±1.6)	−22.3 (±2.0)	−22.6 (±1.7)	−22.1 (±1.9)	0.02	ns
**LV diastolic function**						
E/A	1.37 (±0.28)	1.70 (±0.44)	1.58 (±0.28)	1.70 (±0.34)	ns	ns
E/e’	5.7 (±1.1)	5.7 (±0.8)	5.7 (±1.4)	5.7 (±0.9)	ns	ns
LAS (%)	45.4 (±10.8)	44.5 (±7.9)	47.0 (±6.5)	45.3 (±6.4)	ns	ns
**LV measurements**						
EDD (mm)	41.9 (±7.6)	41.8 (±8.2)	40.9 (±5.2)	40.4 (±4.9)	ns	ns
EDD Z-score median (IQR)	0.3 (−0.93–1.57)	0.37 (−0.47–1.39)	0.54 (0.03–1.03)	0.39 (0.15–0.74)	ns	ns
IVSd (mm)	7.2 (±1.5)	7.2 (±1.4)	6.5 (±0.9)	6.5 (±0.8)	ns	ns
IVSd Z-score median (IQR)	0.73 (0.44–1.18)	0.84 (0.67 –1.22)	0.48 (0.27–0.89)	0.50 (0.22–0.75)	ns	ns
PWd (mm)	7.3 (±1.8)	7.1 (±1.5)	6.5 (±0.8)	6.4 (±0.8)	ns	ns
PWd Z-score median (IQR)	0.32 (0.13–0.71)	0.31 (0.13–0.79)	0.20 (−0.07–0.55)	0.23 (−0.07–0.38)	ns	ns
EDV (mL)	70.3 (±35.7)	71.2 (±33.4)	63.0 (±22.1)	59.6 (±17.8)	ns	ns
EDVi (mL/m^2^)	51.8 (±12.6)	52.9 (±9.0)	55.1 (±7.7)	49.8 (±5.7)	ns	ns
LV mass (g)	95.5 (±45.5)	92.9 (±44.1)	77.3 (±27.3)	70.8 (±25.9)	ns	ns
LV mass indexed for BSA (g/m^2^)	71.0 (±15.3)	68.5 (±14.6)	67.2 (±13.8)	61.2 (±8.9)	ns	ns
LV mass indexed for heigh (g/m)	34.4 (±3.6)	32.1 (±3.0)	34.1 (±4.7)	30.3 (±3.0)	ns	ns

## Data Availability

The data presented in this study are available on request from the corresponding author. The data are not publicly available due to privacy reasons.
